# Glucagon‐Like Peptide‐1 Receptor Agonist‐Based Agents and Body Composition: Filling *More* Gaps

**DOI:** 10.1002/oby.70212

**Published:** 2026-07-08

**Authors:** Robert L. Dubin, Tierra N. Sanders, Hunter M. Schwab, Steven B. Heymsfield, Frank L. Greenway

**Affiliations:** ^1^ Pennington Biomedical Research Center Louisiana State University Health Sciences Baton Rouge Louisiana USA

## Abstract

The last 2 decades have seen historic advances in the treatment of obesity. We are entering an era of defining obesity not by weight‐based outcomes but by changes in body composition. Glucagon‐like peptide‐1 receptor agonists (GLP‐1 RAs) are forming the platform for obesity treatments based on their high level of efficacy. Body composition can be quantified by changes in percent fat/fat‐free mass as a total of body weight. The amount of weight that is lost as % fat‐free mass (% FFML) with diet‐induced weight loss is ~25% but is extremely variable. Our aim was to provide an update to our previous report assessing changes in body composition using GLP‐1 RA‐based agents. In our updated review of 40 DXA‐based reports, we found the mean (SD) % FFML was 29.1% (19.0%). Similar to our previous report, we found that the changes in body composition using these agents remain in the upper range of expected % FFML using other nonsurgical interventions for obesity. Functional measures of strength and performance and assessing body composition effects of future GLP‐1‐based agents are needed to understand the continued gaps that exist in determining the impact these treatments have, particularly in high‐risk populations.

## Study Importance

- What is already known?
  ○ The proportion of weight loss that comes from fat‐free mass using most diet‐induced weight loss interventions is approximately 25%.
  ○ GLP‐1 RA‐based treatments provide substantial amounts of weight reduction and benefits beyond purely weight loss.
  ○ The data regarding the impact that GLP‐1 RA‐based treatments have on body composition remain uncertain.
- What does this review add?
  ○ This updated review of 40 DXA‐based studies and over 2000 participants using GLP‐1 RA‐based treatments suggests that the proportion of weight loss that comes from fat‐free mass is in the upper range of expected amounts of weight loss using similar interventions.
  ○ A high degree of variability exists in currently available studies.
  ○ Many confounding variables exist that contribute to the extremely high degree of variability reported in these studies.
  ○ Beyond surrogate markers of body composition (such as DXA), more clinically relevant outcomes (functional status) are needed to understand the impact that these agents have in treating obesity.
- How might these results change the direction of research or the focus of clinical practice?
  ○ Future studies using GLP‐1 RA‐based treatments should control for confounding factors and assess additional outcomes such as muscle quality and functional outcomes.

## 1 Introduction

The last 2 decades have seen unprecedented progress in the science of obesity medicine. In a recent landmark publication, the Lancet Commission put forth a clinical approach attempting to redefine the disease of obesity by moving beyond body mass index (BMI), shifting from current weight‐based outcomes, and focusing on the disease organ, adipose tissue [1]. Several recommendations made by the commission for estimating the burden of obesity included anthropometric (waist circumference) and imaging modalities bioelectrical impedance analysis (BIA) and DXA (dual‐energy x‐ray absorptiometry) to provide more detailed biomarkers of disease. Emerging technologies such as 3D body composition are being validated in order to measure adiposity and to monitor response to therapy [2].

Aligning with the redefinition of obesity, the treatment landscape based on incretin‐based agents, namely glucagon‐like peptide‐1 receptor agonists (GLP‐1 RAs), has provided additional enthusiasm [3]. What was once a limited landscape of medical options has evolved into a highly efficacious class of hormonal agents providing clinical outcomes that are nearing what was only possible with metabolic bariatric surgery (MBS).

The basis for improved efficacy while using GLP‐1 RAs is their ability to reduce gastric emptying and suppress appetite [4, 5, 6]. Neural pathways in the hypothalamus that control food intake have abundant GLP‐1 receptors [7]. Anecdotal reports have surfaced describing cases of excessive weight loss and malnutrition [8]. Concerns from the lay press have been raised regarding the loss of skeletal muscle while a divergence of opinion exists in the scientific community [9, 10]. Based on limited cohorts reporting outcomes on body composition using these agents, a major gap exists in understanding how these compounds affect body composition. This has led to questioning the clinical impact of these biomarkers of lean tissues while providing substantial improvements in multiple non‐weight‐related outcomes.

The major question posed with the use of GLP‐1 RAs becomes: are the changes in body composition (and skeletal muscle) part of the expected weight loss process (adaptive) or are the effects from the drugs adding to the reductions in lean mass caused by normal aging?

In this narrative review, we provide a complementary update to our previously published report attempting to understand the impact that GLP‐1 RAs have on body composition after treatment for obesity and adiposity‐based chronic diseases (ABCDs) [11]. We also hope to fill even more gaps in our understanding of how these changes relate to important clinical outcomes (see Table 1 for a list of terms used in our review).

**TABLE 1:** Terminology used in text.

Term	Basic description
Incretins	Gastrointestinal hormones: GLP‐1 and GIP
GLP‐1	Glucagon‐like peptide‐1 (hormone)
GLP‐1 RA	Glucagon‐like peptide‐1 receptor agonist (general term given for drugs in class)
GIP	Glucose‐dependent insulinotropic polypeptide (previously glucagon inhibitory peptide)
GRA	Glucagon receptor agonist
Fat‐free mass (FFM)	Lean body mass (or lean soft tissue) and bone minerals (DXA model); a crude skeletal muscle surrogate
% fat‐free mass loss (% FFML)	Percent fat‐free mass loss from an intervention; obtained from DXA = ΔFFM loss (kg)/Δ body weight (kg)
Lean body mass	All DXA non‐fat components excluding bone
Appendicular lean mass	Derived from DXA lean mass and highly correlated with total muscle mass measured by MRI (a skeletal muscle surrogate)
Weight loss composition	Amount of weight loss that is composed of fat mass and fat‐free mass
DXA	Dual‐energy x‐ray absorptiometry
DXA 2‐compartment model	Fat mass and fat‐free mass
DXA 3‐compartment model	Fat mass, lean soft tissue, and bone minerals
ADP	Air‐displacement plethysmography
CT and MRI	Computed tomography and magnetic resonance imaging
MBS	Metabolic bariatric surgery
VLCD	Very low‐calorie diet (usually < 800 kcal/day)
T2D	Type 2 diabetes
Muscle quantity	Amount of skeletal muscle (in kg)
Muscle quality	Ratio of muscle to fat within skeletal muscle
Functional outcomes	Measurements of muscle strength and function

Throughout the review, we will attempt to provide a framework for understanding how body composition is currently being measured. We will use the term percent fat‐free mass loss (or % FFML) in order to provide a metric for measuring the impact that GLP‐1 RAs have on body composition. It is important to realize that this measurement remains a crude surrogate for skeletal muscle and, as our review demonstrates, has fundamental limitations in making clinical determinations.

## 2 Methods/Search Strategy

We conducted a systematic review to evaluate the impact that GLP‐1 RA‐related agents had on body composition, specifically changes in total body weight, fat‐free mass, and lean mass. The following databases were used: PubMed, Ovid MEDLINE, Embase, Cochrane Central Register of Controlled Trials, and ClinicalTrials.gov for completed trials. Date ranges included published reports or abstracts from 2004 to 2025. Key concepts or search words included GLP‐1 RA, fat‐free mass, lean mass, DXA, and body composition. Inclusion criteria: must have used a GLP‐1 RA agent for at least 3 months and included raw DXA data for total weight loss (kg) and changes in fat mass (kg), fat‐free mass (kg), and/or lean mass (kg). Many of the DXA‐based studies that were reviewed for this report used lean soft tissue (LST) mass to account for changes in body composition occurring after treatment. In those cases, we attempted to combine bone mineral content (BMC) with LST to estimate total fat‐free mass. An additional objective of this report was to complement our previously published database (28 reports in our 2024 review) to a total of 40 studies [11].

## 3 GLP‐1 RAs: Current and Pipeline Agents

The currently available and pipeline GLP‐1 RAs are provided in Table 2. From a historic standpoint, the first hormone discovered by Bayliss and Starling in 1902 was a gut‐derived peptide hormone produced by the S‐cells of the duodenum (secretin) [12]. Incretin hormones are derived and released after eating to regulate glycemic control by enhancing insulin secretion. The two main incretin hormones are GLP‐1 (glucagon‐like peptide) and GIP (glucose‐dependent insulinotropic polypeptide). A major expansion has occurred in the incretin‐based agents that were first approved in 2005 (exenatide), followed by longer‐acting agents (liraglutide) and finally the most efficacious agents to date, semaglutide and tirzepatide (dual GLP‐1 and GIP). Both subcutaneous injectable and oral agents are now available or are in development.

**TABLE 2:** GLP‐1 RA current and pipeline agents.

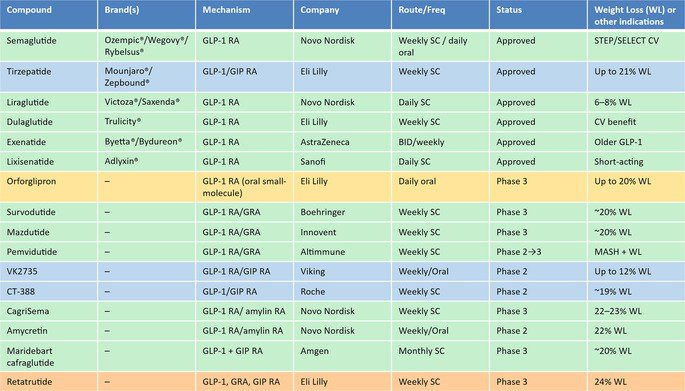

Abbreviations: BID, twice daily; CV, cardiovascular; GIP RA, glucose‐dependent insulinotropic polypeptide receptor agonist; GLP‐1 RA, glucagon‐like peptide‐1 receptor agonist; GRA, glucagon receptor agonist; MASH, metabolic dysfunction‐associated steatohepatitis; SC, subcutaneous.

While GLP‐1 RAs were originally developed for treating type 2 diabetes (T2D), additional clinical benefits, including significant weight reduction for treating obesity and ABCDs, were soon realized. Currently, tirzepatide is the only dual agonist approved for use in the United States. Additional combinations of GLP‐1 RAs are currently in development including a glucagon receptor agonist (GRA), an amylin receptor agonist (ARA), and a polypeptide Y (PYY) agonist combination agent; a combination GLP‐1 RA and GIP antagonist and a triple agent (GLP‐1 RA, GRA, and GIP RA) is also completing Phase 2 and 3 trials [13, 14, 15, 16, 17, 18]. While these and many other treatments are being developed for obesity, it is imperative to understand the impact that these future agents have on body composition (based on their unique hormonal profiles) and take the necessary steps to assess for any untoward safety signals.

## 4 Body Composition Theory

**FIGURE 1:**
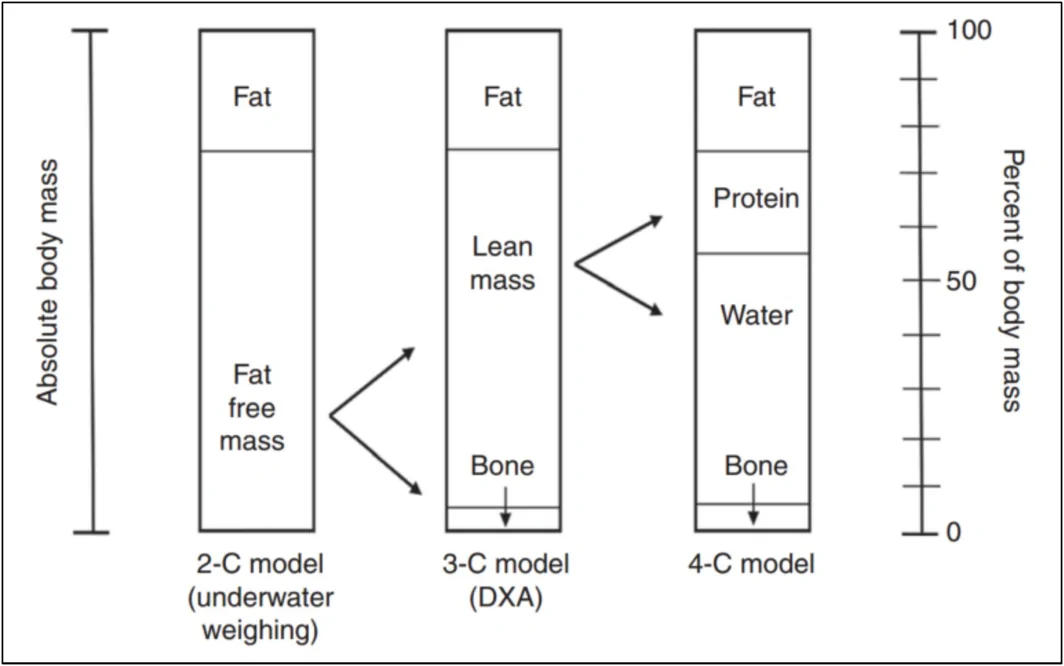
Body composition measurements based on 2‐, 3‐, and 4‐compartment models [19]. DXA, dual‐energy x‐ray absorptiometry.

Body composition analysis enhances the diagnosis of obesity and the monitoring of treatment response. The 3‐compartment body composition model DXA measures fat mass (FM), lean soft tissue (LST), and bone mineral content (BMC) (Figure 1). LST mass includes muscle, organs, blood, and connective tissues and body water and is critical for preservation during weight loss interventions. The amount of LST that includes skeletal muscle is variable (~50%–60%) and is impacted by sex, age, amount of weight loss, and fitness level [20, 21]. The current diagnostic term fat‐free mass (FFM), used in a 2‐compartment model, is equal to total body mass (TBM) minus FM and includes BMC. For the purpose of this report, we will use the term FFM when describing this portion of body composition knowing they are related but not synonymous.

We also use the term % loss of fat‐free mass (% FFML) to equal the percentage of total weight loss due to FFM in describing the impact that treatments have on body composition. We have used this term, consistent with previous reports, to provide an expected amount of FFM loss after treatment and to provide an intuitive reference for reporting changes in body composition. To our knowledge, there is no standard outcome or reference for reporting body composition (absolute vs. relative, regional vs. whole body, lean vs. fat vs. bone, rate of change, and follow‐up duration) after clinical treatments. This is important since authors have reported the changes that occur in body composition with GLP‐1 RAs using outcomes such as total percentage loss of whole‐body FM and FFM or total change (kg) in FM and FFM over time [22, 23].

## 5 Assessment of Body Composition

**FIGURE 2:**
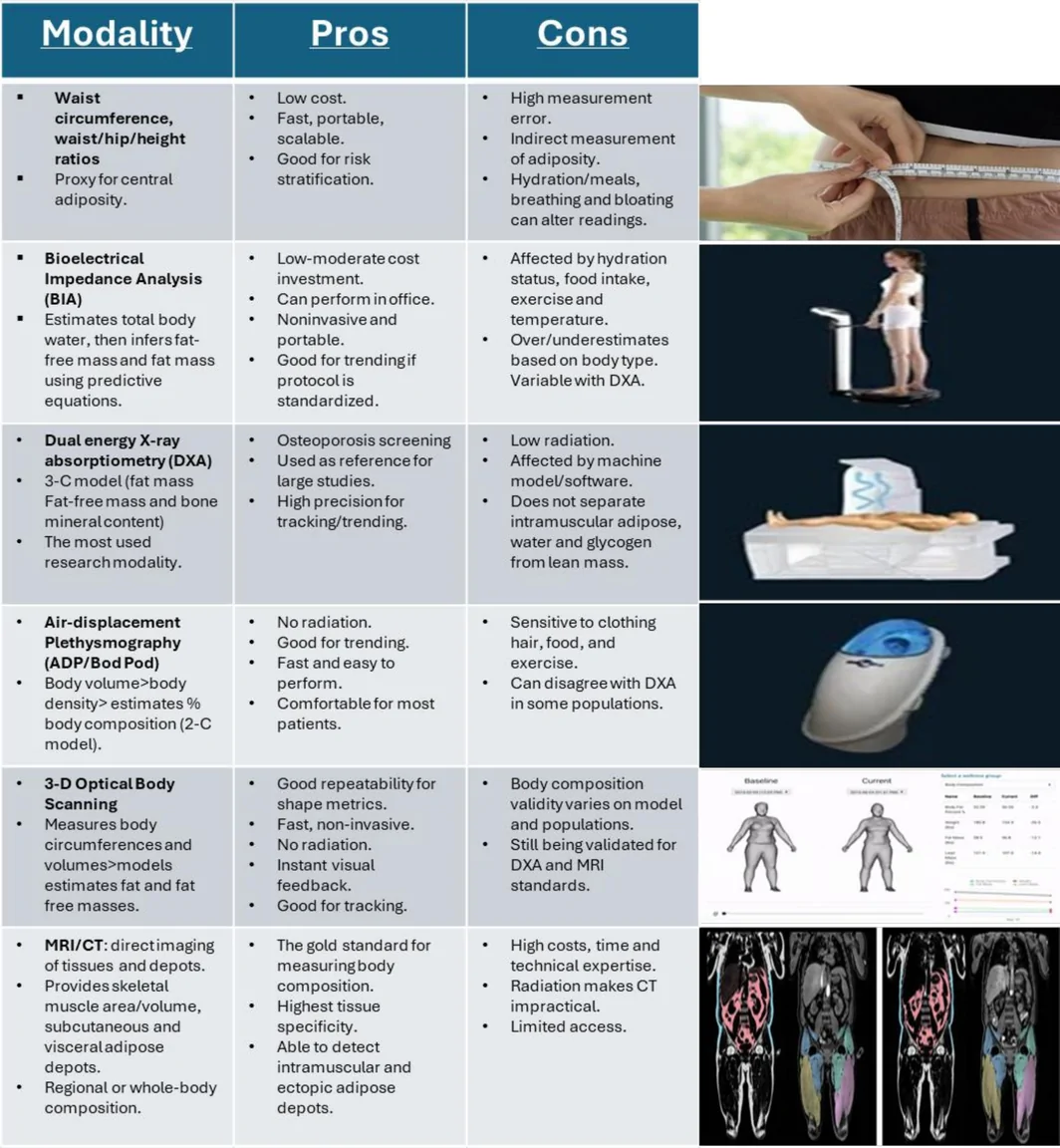
Body composition measuring modalities, including waist/hip/height circumferences and ratios, bioelectrical impedance analysis (BIA), dual‐energy x‐ray absorptiometry (DXA), air‐displacement plethysmography (ADP/Bod‐Pod), 3D body composition, magnetic resonance imaging (MRI), and computed tomography (CT).

Assessing body composition after weight loss requires basic knowledge of body composition methods. There are various ways for measuring body composition in adults [24]. MRI and CT scanning remain the gold standards for directly quantifying and qualifying adipose tissue and skeletal muscle, but they are limited to high‐level research settings [25]. Air‐displacement plethysmography (ADP/Bod‐Pod) is also considered an accurate method for measuring non‐regional body fat and FFM [26]. An additional method for estimating whole‐body skeletal muscle mass for research purposes is D3‐creatine dilution [27, 28]. Additional methods for estimating body composition in clinical practice today, including BIA and anthropometric measurements (waist/hip measurements, BMI, and fat pad measurements), are easy to implement in the office but are not sensitive for determining small changes in body composition and may be affected by body hydration status [29]. An emerging technology known as 3D body composition is available commercially and is under investigation for multiple clinical applications [2] (see Figure 2 for body composition modalities).

In order to achieve consistency in data reporting, we chose to evaluate studies using DXA (and ADP) to determine body composition, since MRI‐ and CT‐based studies during weight loss with GLP‐1 RA‐based agents are scarce. Out of 40 datasets used in our review, we included 4 that used ADP measuring systems [5, 23, 30]. One report used CT imaging since it provided whole‐body changes that were comparable to the DXA dataset [31]. Two additional MRI‐based reports did not provide % FFML as an outcome but are included in order to demonstrate the direct impact of these treatments on skeletal muscle [32, 33].

The DXA 3‐compartment model has been shown to be comparable to MRI and CT for estimating adipose and skeletal muscle mass [34, 35, 36, 37]. It is critical to realize the limitations of DXA for the purposes of measuring body composition. DXA does not measure skeletal muscle mass directly, but instead measures LST mass from all extremities, and the total appendicular lean mass (ALM) correlates with total muscle mass measured from MRI [38, 39]. An additional limitation when using DXA for determining body composition is that it is unable (without additional analytical software) to separate adipose tissue that resides in ectopic regions such as skeletal muscle and bone (intramuscular or bone marrow adipose tissue) [40].

## 6 Effect of Obesity Treatments on Body Composition

The impact of various obesity treatments on body composition can serve as a reference point to determine an expected amount of % FFML from a weight loss intervention, knowing that (in a 2‐compartment model) total weight loss (kg) = total loss of FM (kg) + total loss of FFM (kg). The % FFML will equal total loss of FFM (kg)/total weight loss (kg) × 100. Based on body composition changes that have occurred after other non‐GLP‐1 RA‐based treatments, we will be able to compare expected amounts of % FFML using the 40 studies included in this report.

The amount of FFM that is lost using diet‐induced calorie restriction is highly variable and influenced by various factors (age, sex, diet, exercise, weight loss magnitude and velocity, initial body composition, and hormonal status). There appears to be a consensus that weight loss under standardized conditions should result in at least 75% FM loss and no more than 25% from FFM, termed the “one‐fourth rule to weight loss” [41]. MRI‐based studies have also demonstrated that under tightly controlled calorie restriction and physical activities, the percentage of skeletal muscle loss in women and men should equal 10%–15% and 20%–25%, respectively [42].

A predictive model that was proposed in 1987 (the Forbes rule) described a linear relationship that exists between body composition after small weight losses that was dependent on initial FM. Using these equations, the % FFML predicted with most diet‐induced weight loss interventions (5%–10% total weight loss) would be ~25% [43].

The impact of significant (over 20% of baseline weight) and rapid weight loss on body composition, such as using very low‐calorie diets (VLCD), indicates a high degree of variability based on the aforementioned factors [19, 44]. After losing over 50% of their baseline weight while maintaining an adequate protein intake and a minimum of 2 h per day of high‐intensity resistance training, participants still lost 20% of their weight from FFM at 30 weeks using DXA measurements [45].

The effect of MBS on body composition with DXA‐based studies has demonstrated % FFML between 20% and 30%, with Roux‐en‐Y gastric bypass (RYGB) producing the greatest loss [46] and with over half of % FFML occurring rapidly within the first 3 months postoperatively [47]. Additional reports demonstrate that at 1 year post RYGB, females losing 35.2% of their total baseline weight had only 16.6% FFML (DXA), while the loss of skeletal muscle (as a percentage of total weight loss) with whole‐body MRI was reported at 11.1% [46]. The report concluded that % FFML during the first year was almost entirely (96.7%) skeletal muscle.

In line with our initial report of GLP‐1 RA‐based body composition [11], the new analysis is from small clinical trials (mean *N* = 51 ± 92 participants). For context, we will provide examples from the 40 reports (from 2004 to 2025) that used DXA/ADP imaging (1 report used whole‐body CT) for assessing changes in FM and FFM.

The mean for total weight loss of the cohort was −7.0 ± 7.3 kg. Total changes in FM and FFM were −4.3 ± 5.7 kg and −1.7 ± 3.0 kg, respectively. The mean % FFML for all studies was 29.1% ± 19% (Table 3). The following examples demonstrate the heterogeneity that exists in our data:

**TABLE 3:** All studies included in review.

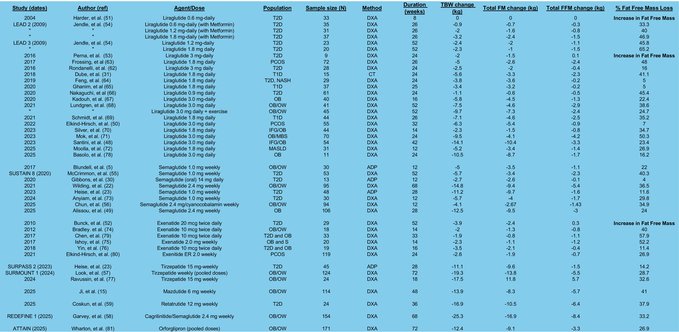

Abbreviations: ADP, air‐displacement plethysmography (BOD‐POD); CT, computed tomography; DXA, dual‐energy x‐ray absorptiometry; IFG, impaired fasting glucose; MASLD, metabolic dysfunction associated liver disease; NASH, nonalcoholic steatohepatitis; PCOS, polycystic ovarian syndrome; S, schizophrenia; T1D, type 1 diabetes; T2D, type 2 diabetes; TBW, total body weight.

In a Swiss observational study by Santini and associates, with liraglutide 3.0 mg daily for 10 months in 54 participants with obesity, % FFML was 23.4% [48]. Alissou and colleagues reported in a cohort of 106 participants with obesity and comorbidities % FFML of 24% using semaglutide 2.4 mg weekly for 28 weeks using DXA imaging [49]. In a cohort of 55 participants with polycystic ovarian syndrome (PCOS), treatment with liraglutide 3.0 mg daily for 32 weeks provided DXA % FFML of only 7% [50]. In a report of 15 participants with T2D treated with once daily oral semaglutide for 12 weeks, participants lost a total of 2.7 kg with only 4.0% accounted for as % FFML measured by ADP [30]. Three studies from our analysis resulted in increases of FFM after weight loss using GLP‐1 RAs [51, 52, 53].

Data from the LEAD‐2 trial (103 participants with T2D) using liraglutide for 1 year at 0.6, 1.2, or 1.8 mg daily demonstrated a dose‐dependent % FFML of 33.3%, 40.0%, and 46.9%, respectively [54]. In the SUSTAIN 8 trial reporting 53 participants with T2D treated with semaglutide 1 mg weekly for 52 weeks, participants experienced a loss of 40.4% of their total weight loss from FFM using DXA [55]. An additional report, using compounded semaglutide/cyanocobalamin in an obesity cohort (*N* = 94), demonstrated % FFML of 34.2% after 3 months of treatment [56].

The dual‐incretin tirzepatide reduced FFM by 14.2% (total 11 kg weight loss) after 7 months in 48 participants with T2D compared to 11.6% FFML (total 7 kg weight loss) using low‐dose semaglutide in 45 participants using ADP/Bod‐Pod analysis [23]. The SURMOUNT 1 trial (pooled tirzepatide doses in participants with T2D) found % FFML of 28.7% at 72 weeks [57].

## 7 Additional Reports and Body Composition

In evaluating the combination amylin/GLP‐1 RA (CagriSema) when used to treat obesity, in a sample of 154 participants, DXA measurements at 68 weeks demonstrated % FFML of 33.2%, while a study with the GLP‐1 RA/GRA mazdutide in Chinese adults reported % FFML of 41.0% at 48 weeks [14, 58]. A study with the triple agent retatrutide in a small sample (*N* = 24) of patients with T2D found that 37.9% of the total weight loss (at 36 weeks) was from FFM, maintaining a comparable degree of % FFML as previously reported with single GLP‐1 RAs [59].

A meta‐analysis (using DXA) assessing the impact of GLP‐1 RA‐based agents (liraglutide, exenatide, semaglutide, and dulaglutide) on body composition in participants with T2D and obesity was conducted by Sargeant et al. in 2019 [60]. They reported similar outcomes in body composition using GLP‐1 RA and sodium glucose transport inhibitor‐2 (SGLT2) agents. The % FFML from their review was quite variable (20%–50%) and was felt to be consistent with other forms of weight loss (diet‐induced weight loss or MBS).

An additional DXA meta‐analysis from Beavers and associates determined that approximately 30% of body weight lost with GLP‐1 RAs was from lean mass [61]. A compilation of all studies that were reviewed are listed in Tables 3 and 4 [5, 15, 22, 23, 30, 31, 48, 49, 50, 51, 52, 53, 54, 55, 56, 57, 58, 59, 62, 63, 64, 65, 66, 67, 68, 69, 70, 71, 72, 73, 74, 75, 76, 77, 78, 79, 80, 81].

**TABLE 4:** % fat‐free mass loss (%FFML) from each study reported in review.

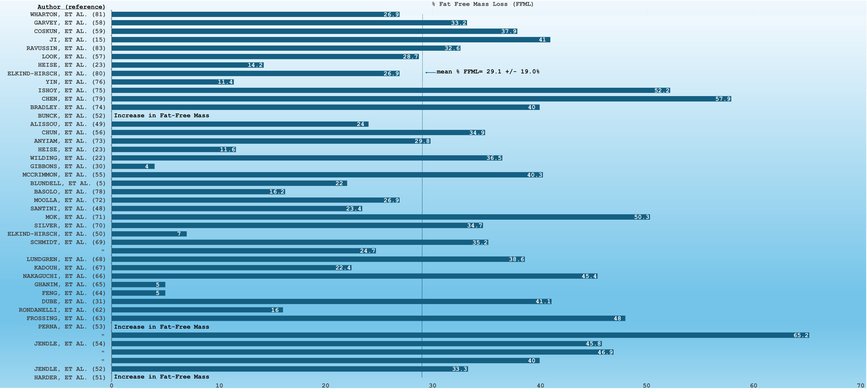

## 8 Caveats to Reported Studies

There were numerous reasons why the reports reviewed for this analysis demonstrated such heterogeneity: extremely small samples and lack of control for confounding factors are clearly a cause for concern. No studies controlled for baseline weight/body composition status, amount/velocity of weight loss, and follow‐up time, and all that can impact changes in body composition. The mean time for testing body composition in our review was 30 ± 18 weeks but varied from 12 weeks to over 1 year. There is no consensus in time for testing body composition occurring after weight loss.

Under normal physiologic conditions, there is no way to completely avoid losing some amount of FFM when undergoing a weight loss intervention. Even when using a ketosis‐based (low‐carbohydrate/high‐protein) diet, after hepatic glycogen stores have been exhausted (1–2 days), gluconeogenesis (2–5 days) provides the primary fuel source converting proteins to glucose for brain survival. Reductions in mechanical loading, amino acid intake, and hormonal changes then all contribute to increased muscle catabolism making the loss of FFM inevitable.

Hall revised the initial Forbes rule in 2007 in predicting the changes that would occur in body composition with larger degrees of weight change, noting that the original Forbes rule underestimated % FFML with moderate‐large degrees of weight loss. He used a nonlinear equation that included the magnitude and direction of weight changes in addition to initial FM to predict the changes that would occur after weight loss/gain [82]. His model suggests, based on the magnitudes of weight loss with GLP‐1 RAs, similar amounts of % FFML as our cohort suggests (20%–30%). The mean total weight loss in our cohort was only −7.0 ± 7.3 kg with the total change in FFM of only −1.7 ± 3.0 kg, so absolute changes in body composition were relatively small.

The other major limitation to our analysis is that DXA does not measure skeletal muscle directly nor does it account for ectopic adipose depots such as skeletal muscle (intramuscular adipose tissue), bone (bone marrow adipose tissue), or liver (intrahepatic adipose tissue) that may be the primary sites for early and rapid weight loss. The presence of adipose tissue in these (FFM) organs would overestimate the amount of % FFML that is reported by DXA.

Is there a difference with the single GLP‐1 RA agents on body composition or does the dual‐incretin GLP‐1/GIP have a more favorable impact? Recent reports from Ravussin and colleagues indicate that the dual‐hormone tirzepatide increases fatty acid oxidation which may improve fat to lean mass ratios [77]. Although the mechanisms for additional GLP‐1 RA therapies including GRA, GIP, and ARA may have direct and indirect effects on body composition, the data are not yet available to make firm conclusions.

Another factor in assessing the reports of GLP‐1 RAs on body composition is based on the presence of T2D in the participants who are studied. In the 40 datasets we included, only 1 reported subgroup analysis, demonstrating less favorable responses in body composition in participants with T2D [49].

Studies have also indicated a consistent weight loss outcome advantage for women over men using GLP‐1 RAs [83, 84]. Women are known to have higher baseline FM body composition and account for a majority of participants in clinical obesity trials. According to the Forbes rule, men (who are leaner) should lose more absolute FFM when losing weight, but none of the reports that were included in this review provided sex‐stratified body composition outcomes.

Reporting how the results are expressed is another way to understand the current body composition reports using GLP‐1 RA agents. We chose to use the outcome % FFML as an indicator describing the proportion of the total weight loss that was from FFM (or lean mass). This is in line with our previous report documenting body composition with GLP‐1 RA‐based interventions [11]. If using % FFML provides a benefit over other types of outcome reporting, it is unclear but was based on the available data and provides an intuitive metric.

There are aspects of assessing body composition that require a greater understanding of clinically relevant endpoints such as functional testing (strength), cardiorespiratory function (VO2 max), and the occurrence of falls/injuries, bone fractures, and mortality. A report from Alissou and associates indicated that functional status using handgrip strength increased significantly (+ 4.5 kg) at 12 months after 28 weeks of semaglutide in participants with obesity and comorbidities [49]. In a report of 23 patients with T2D, there was no improvement in exercise capacity as assessed by peak oxygen consumption (VO2 max) after 3 months of exenatide 10 mcg BID [85].

**FIGURE 3:**
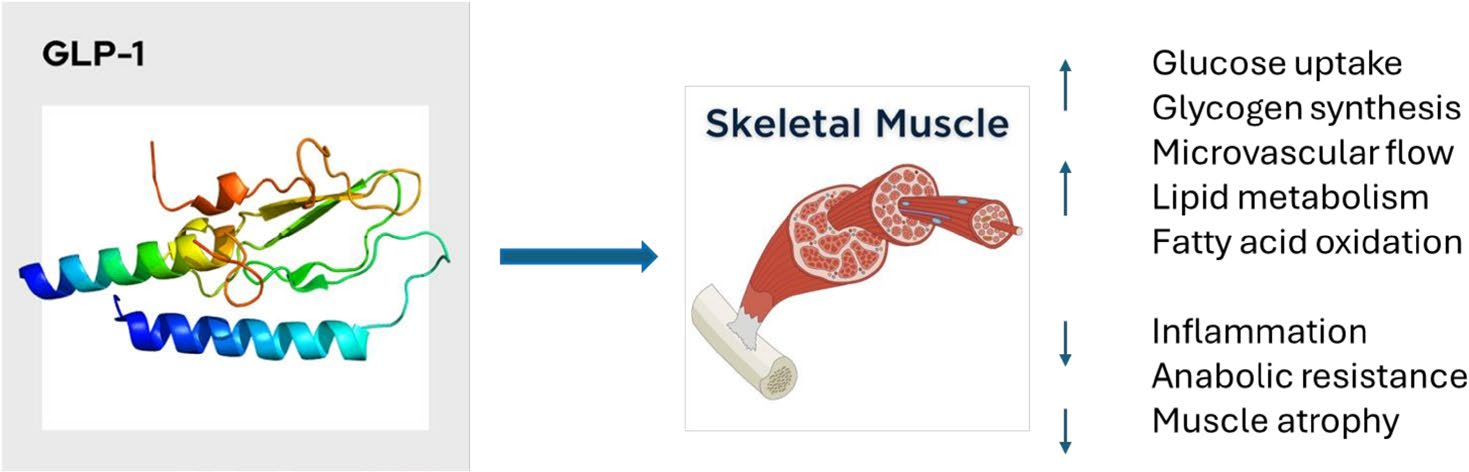
Endogenous effects of GLP‐1 hormone on skeletal muscle.

An additional aspect of understanding how GLP‐1 RA‐based agents may impact body composition is knowing how the endogenous GLP‐1 hormone acts on skeletal muscle. The available data show that GLP‐1 hormone has a consistent positive influence on supporting skeletal muscle (Figure 3) [86, 87, 88].

## 9 What Is Our Best Response to GLP‐1 RA‐Based Agents and Body Composition?

Studies supporting measures to enhance body composition when using GLP‐1 RA‐based agents are needed to determine the best supportive care for our patients. One such report using GLP‐1 RAs that included a resistance exercise arm was from Lundgren and associates. Participants randomized to liraglutide 3.0 mg daily plus exercise (mainly aerobic) for 1 year had less (24.7% vs. 38.6%) relative % FFML versus drug therapy alone [68].

Another potential option is to slow the weight loss trajectory using these agents. In a recent report from Eldor and associates, a gradual dose titration of semaglutide (i.e., microdosing) resulted in fewer adverse events and improved treatment adherence versus a label‐recommended titration regimen [89]. If this approach has any impact on preserving FFM using these agents remains unanswered.

An emerging area of research that deserves special mention for improving body composition while using GLP‐1 RAs is muscle‐preserving agents including the myostatin‐activin pathway inhibitors (MAPi) and selective androgen receptor modulators (SARMs). New compounds such as bimagrumab (Lilly), trevogrumab/garetosmab (Regeneron), and taldegrobep (Biohaven), which inhibit myostatin (and or activin signaling), show promise in preventing muscle loss while promoting fat reduction for treating obesity with GLP‐1 RAs [90, 91, 92].

Bimagrumab is currently in trials combined with semaglutide (SEMA) to assess its potential for preserving muscle mass during weight loss. Preliminary results have demonstrated that 92.8% of the total weight loss using bimagrumab/SEMA was from FM. Similarly, Regeneron plans to release Phase 2 data on trevogrumab and garetosmab designed to preserve and possibly increase FFM with use of GLP‐1 RAs. For another agent, enobosarm (Veru), a SARM, top‐line results (unpublished) were released in a study of 168 participants (> 65 years) with obesity who all received SEMA and either 3 or 6 mg plus enobosarm or placebo for 16 weeks. The average weight loss (approximately −4.5 kg) was similar for both placebo and pooled enobosarm/SEMA doses. However, the percentage of lean mass of the total weight loss was 31.9% (placebo/SEMA) versus 9.4% for enobosarm/SEMA. Functional status (stair climb power) loss was greater with placebo/SEMA versus the enobosarm/SEMA group.

Assessing outcomes beyond muscle mass using these and similar agents is critical. In a recent meta‐analysis, bimagrumab treatment significantly increased thigh muscle volume (by 5.3%) and FFM (+1.9 kg) while decreasing FM versus placebo (−4.6 kg). However, no significant improvement was seen in muscle strength or physical performance (gait speed or 6‐min walk test) with treatment, except among those with a slower walking speed or distance at baseline [93].

## 10 What Does the Future Hold?

Based on the significant advances that have occurred in the last 2 decades in treatment efficacy and identifying tissue‐specific targeted therapies, the future holds great hope as well as challenges. Keeping in step with this historic trajectory will require making improvements in the tolerability and durability of the currently expanding pharma‐based agents as we close the current medical‐surgical treatment gap. Improving weight loss quality by enhancing the ratio of FM/FFM loss will be necessary to accurately identify the changes that occur while using these agents. We have made the argument that the current DXA‐based methods for making these determinations have limitations that may be contributing to the heterogeneity existing in the current analysis. The limitations that most DXA systems have are based on the inability to measure intermuscular adipose tissue that may be a primary target of rapid and large‐volume weight loss, thereby overestimating the amount of % FFML. If this is taken in consideration with the mean % FFML (29.2% ± 16.2%) seen in our current cohort, we may near the anticipated changes (25%) in FFM based on similar magnitudes of weight loss.

The use of advanced imaging such as MRI appears to be a plausible solution to the high variability that is inherent with DXA‐based studies. One report from a subgroup analysis of the SURPASS‐3 MRI study revealed the dual‐incretin tirzepatide reduced muscle fat infiltration (MFI) by 4.4% versus controls in participants with T2D after 52 weeks of treatment [32]. Another MRI‐based substudy from Pandey et al. assessed muscle quality in 128 females with obesity and no diabetes. They found that compared to placebo, liraglutide reduced (thigh) muscle fat by 7.8% from baseline, and that the presence of adverse muscle composition (high muscle fat/low muscle mass) decreased compared to controls [33]. Is it possible that we have been using limited measuring techniques (DXA) for accurately assessing body composition in this new era of interventions? In addition to measuring muscle quantity with DXA (% FFML), we should also be measuring muscle quality (amount of muscle fat and adverse muscle composition) with MRI, at least in research‐level subgroup analysis. While we await further answers, emerging techniques such as 3D imaging and D3‐creatine hold promise for the future in larger‐size clinical trials [2, 27, 28].

Another caveat for the future is the role of muscle‐preserving agents such as MAPi and SARM compounds. Are we making major presumptions to improve body composition with current GLP‐1 RA platforms before understanding the overall effect that these compounds have on body composition and function? The fact that such agents may not improve functional and performance outcomes (regardless of improvements in body composition) is a potential cause for concern. This all provides an opportunity to return to clinical fundamentals through lifestyle modifications (protein‐enhanced diets and resistance‐based exercises) as foundational treatments for high‐quality obesity interventions.

The recommended clinical approach is to identify high‐risk individuals (elderly and postmenopausal females) who would benefit from closer monitoring and more intense mitigation strategies. This requires a more extensive diagnostic and treatment approach while managing these patients to ensure we are meeting important non‐weight‐related outcomes. The use of office‐based anthropometrics/BIA and DXA in high‐risk patients when assessing body composition and bone health should be part of our current strategy. Functional testing such as sit to stand, handgrip, and walk testing can be added to provide additional clinical measures while determining outcomes for cardiorespiratory fitness as an additional research‐level outcome.

The future holds great promises for the new era of obesity medicine. A recent statistical analysis suggests we are starting to see improvements in reducing obesity rates in the United States [94]. The addition of mandatory nutrition‐based and lifestyle training through medical education is gaining traction [95]. Obesity medicine fellowships and the American Board of Obesity Medicine (ABOM) are now providing a critical subspecialty to provide the high level of management required to treat this complex disease [96]. The United States federal government is assessing options of increasing access to obesity treatments for Medicare recipients which will require improving return on investment for such costly treatments [97].

In pondering the question of whether GLP‐1 RA‐based agents cause excessive loss of muscle and vital tissues and what, if any, is the clinical relevance, it is best to remember the words of Sir William Osler, who wrote of the benefits of maintaining equanimity as one of the greatest character traits for all physicians [98]. One would suspect if there was a concerning safety signal from the effect of these drugs on body composition after over 20 years of usage (in T2D), we would have identified such a safety signal. In fact, the opposite is true in case of cardiovascular and ABCD benefits. While these critical questions await further confirmation, we will continue to do our part redefining the disease of obesity, closing the medical‐surgical chasm, and filling even more gaps that exist in our current understanding.

## Funding

The authors have nothing to report.

## Conflicts of Interest

R.L.D. was on the Lilly Speaker Bureau for 2025. T.N.S. and H.M.S. declare no conflicts of interest. S.B.H. serves on the Medical Advisory Boards of Tanita Corporation, Novo Nordisk, Abbott, Regeneron, and Medifast. F.L.G. holds advisory positions for Altimmune Inc., Rejuvenate Bio, and Energesis Pharmaceuticals, and he is a shared investor with Melior Pharmaceuticals, Nov‐Meta Pharmaceuticals, and Biohaven Pharmaceuticals.

## Data Availability

The data that support the findings of this study are available from the corresponding author upon reasonable request.
